# Gender differences in characteristics of violent and sexual victimization in patients with psychosis: a cross-sectional study

**DOI:** 10.1186/s12888-021-03558-8

**Published:** 2021-11-01

**Authors:** E. C. D. van der Stouwe, L. A. Steenhuis, G. H. M. Pijnenborg, B. de Vries, Pharmacotherapy and outcome survey (PHAMOUS)-investigators, A. A. Bartels-Velthuis, S. Castelein, W. Veling, E. Visser, J. T. van Busschbach

**Affiliations:** 1 University of Groningen, University Medical Center Groningen, University Center of Psychiatry, Groningen, The Netherlands; 2 GGZ-Drenthe, Assen, The Netherlands; 3 Department of Clinical & Developmental Neuropsychology, University of Groningen, Groningen, the Netherlands; 4 Lentis Psychiatric Institute, Groningen, The Netherlands; 5 Department of Clinical Psychology, University of Groningen, Groningen, the Netherlands; 6 Department of Human Movement and Education, Windesheim University of Applied Sciences, Zwolle, The Netherlands

## Abstract

**Introduction:**

Various studies have demonstrated that individuals with a psychotic disorder are at an increased risk of becoming a victim of crime. Little is known about gender differences in victimization types and in specific characteristics of victimization (e.g., perpetrator, location or disclosure). Knowledge on characteristics of victimization would provide clinicians with more insight which may be especially useful for tailoring interventions. The aim of this study is to examine gender differences in characteristics of violent and sexual victimization in patients with a psychotic disorder.

**Methods:**

Information on violent (threats, physical abuse) and sexual victimization (harassment, assault) was assessed in 482 individuals with a psychotic disorder who received mental health care. Patients were recruited through a routine outcome monitoring study and a clinical trial.

**Results:**

Men reported more threats with violence (20.7% vs. 10.5%, x^2^ = 7.68, *p* = 0.01), whereas women reported more sexual assault (13.3% vs. 3.6%, x^2^ = 15.43, *p* < 0.001). For violent victimization, women were more likely than men to be victimized by a partner, friend or family member (52.9% vs. 30.6%) as opposed to a stranger (11.8% vs. 40.3%; O.R. = 52.49) and to be victimized at home (60.0% vs. 29.3%) as opposed to on the street or elsewhere (40.0% vs. 70.3%; O.R. = 0.06). For sexual victimization, there was no difference in location and perpetrator between men and women. For sexual victimization and physical violence, no differences in disclosure were found, but women were more likely not to disclose threats with violence or to disclose threats to a professional or police (52.9% vs. 45.2%; O.R. = 30.33). All analyses were controlled for age, diagnosis and employment.

**Discussion:**

Gender patterns of victimization types and characteristics are similar for individuals with a psychotic disorder in comparison to the general population. Men were at higher risk of violent victimization, whereas women were at higher risk for sexual victimization. Men were more likely to become victimized in the streets or elsewhere by a stranger, whereas women seemed to be more often victimized at home by a partner, friend or a family member. Future studies may tailor interventions preventing victimization in psychosis according to gender.

## Introduction

Individuals diagnosed with a psychotic disorder are at an increased risk of becoming a victim of crime. According to a meta-analysis on victimization in this population, the median prevalence rate during adulthood is 66% for violent victimization (e.g., physical assault, threat with violence or with a weapon) and 27% for sexual victimization (e.g., forced sexual penetration, sexual touch without consent, or sexual harassment) [1]. Patients with a psychotic disorder are therefore approximately four to six times more likely to become a victim of a crime compared to the general population [2, 3]. Victimization is a highly stressful occurrence and may negatively affect multiple domains of life, including occupational functioning and intimate relationships [4]. In addition, victimization in patients with psychosis is associated with more severe symptomatology, increased use of psychiatric services, poorer illness outcome [5], and also elevated risk of re-victimization [6]. The prevalence of victimization in psychiatric patients has been the topic of several studies in the past two decades. However, only a few studies reported prevalence rates of different types of victimization disaggregated by gender [1], and no previous study has investigated gender differences in specific characteristics (e.g., perpetrator, location or disclosure) of different types of victimization (e.g., violent, sexual) in patients with a psychotic disorder.

With regard to prevalence rates of different types of victimization in the general population, men are consistently found to be at elevated risk of violent victimization [7, 8] and women are more often victim of sexual violence [7, 9]. This clear gender pattern has not been consistently found in patients with a psychotic disorder [10]. reported an odds ratio of 1.55 for violent victimization of women with psychosis compared to men and similarly, [11] reported significantly more physical (56% vs. 12%) but also sexual (40% vs. 2%) victimization in women with schizophrenia compared to men. However, [12] reported more violent victimization in men (O.R. = 3.93) than in women with a psychotic disorder. Lastly, several studies found no association between violent and/or sexual victimization and gender in people with a psychotic disorder [3, 8, 13, 14]. It is therefore still unclear how gender affects victimization rates in different types of victimization in psychosis, so more research is needed to resolve inconsistent findings.

In addition to clarification of gender differences in victimization type prevalence, it may be especially relevant to gain insight in gender differences in the characteristics of different types of victimization. In the general population, men are more often victimized by a stranger and women are more often victimized by a friend or an intimate partner [7, 9]. With regard to location, men are more likely to be victimized in public space, whereas women are more often victimized at home [7]. Around 44% of violent victimization incidents are reported to the police, and this does not differ between men and women (CBS, 2015 [15];. Notably, the aforementioned studies do not distinguish between different types of victimization (e.g. violent or sexual) when examining characteristics. In a recent clinical study in patients with a dual diagnosis a similar trend was found as in the general population [16]: more men reported violent victimization in public space by a stranger, whilst more women reported violent victimization at home by an (ex)partner. However, with regard to disclosure, there was no significant gender difference in speaking with others about the incident but men with dual diagnosis were less likely to report physical victimization to the police compared to women [16].

The aim of the current study is to investigate gender differences in the prevalence of different types of victimization and in characteristics of violent and sexual victimization, in patients with a diagnosis in the psychotic spectrum. Characteristics such as the type of perpetrator, location, reporting to the police and speaking with others about recent violent and sexual incidents will be explored. Knowledge on which characteristics of victimization are influenced by gender provides clinicians with more insight in victimization which is often overlooked, and can be especially useful for tailoring preventive interventions. Consistent with studies in the general population, it is hypothesized that men with psychosis report more violent victimization and women with psychosis report more sexual victimization. Based on studies on ‘overall’ (that is, without distinguishing between violent and sexual) victimization in the general population, we hypothesize that men are most often victimized in public by strangers and that women are most often victimized by partners or familiar people at home for the different victimization types. No gender differences in disclosure are expected.

## Methods

### Sample and procedure

In the current study, we combined data from three separate studies. Baseline data from the pilot study [17] and the multi-centre randomized controlled trial [18] of the BEATVIC project were used. The BEATVIC project aimed to gain more insight in victimization of individuals with psychosis and to develop and investigate the efficacy of a body-oriented preventive intervention. Additional data were extracted from a large ongoing Dutch observational cohort study, the Pharmacotherapy Monitoring and Outcome Survey (study 3; PHAMOUS [19];. For PHAMOUS, patients with psychotic disorders from four mental health institutions in the northern Netherlands are assessed yearly using Routine Outcome Monitoring (ROM), which is part of regular clinical practice. For a sub-study within this monitor, the VICTROM study, a victimization questionnaire was added to the screening. The protocols of the three aforementioned studies were approved by the medical ethical board of the University Medical Center Groningen and conducted in accordance with the Helsinki Declaration guidelines. For each study, participants provided written informed consent to participate and consent for processing personal data for research purposes. Before giving consent, participants were informed that a potential disadvantage of participating in the study could be that questions might be experienced as difficult or confronting.

### Participants

In the current study, individuals with a diagnosis in the psychotic spectrum according to DSM-IV [20] or DSM 5 [21] who were in care at a mental health institution, were included in the analyses. Participants had to be 18 years of age or older and be able to provide informed consent. For BEATVIC [18] exclusion criteria consisted of severe psychotic symptoms, substance dependence, co-morbid neurological disorder or personality disorder, IQ below 70 or pregnancy. For PHAMOUS [19] no further exclusion criteria were utilised.

### Measures

#### Demographic information

Patients completed demographic questions regarding age, gender, occupational status and living situation. For the purpose of this study, occupational status was categorized into no work, less than 12 h of work per week or more than 12 h of work per week. Living situation was conceptualised as independent living (with or without others) or supported housing (formal support available 24/7).

#### Positive and Negative Symptoms

All participants completed the Positive and Negative Syndrome Scale (PANSS [22];. The PANSS is a semi-structured interview which assesses symptom severity of positive symptoms, negative symptoms and general symptoms. Symptoms were conceptualised as a frequency score of total (30 items), positive (7 items), negative (7 items) and general symptoms (16 items). Higher scores indicate a higher frequency of symptoms.

#### Victimization

Victimization was assessed with the Safety Monitor, a victimization survey that is the Dutch equivalent of the International Crime Victims Survey (ICVS; 23). The Safety Monitor is used by the governmental institution Statistics Netherlands (In Dutch: CBS) to measure victimization on a large scale and has often been used to study victimization in patients with severe mental illness and dual diagnosis [2].

The Victimization subscale [23] of the Safety Monitor was used to examine whether participants experienced different types of victimization in the past twelve months and in the past 5 years. In this study, data regarding threats of violence and actual physical assault (violent victimization) and sexual harassment and assault (sexual victimization) were used. In case a certain type of victimization was reported, characteristics of the most recent incident were further explored by means of questions regarding the perpetrator (stranger, partner, friend, family, acquaintance, neighbour, other patient), the location (home, street, elsewhere), and whether the participant had spoken about the incidence to others and to the police (no one, partner, family, friend, professional, police). Due to the large number of details per incident some levels of categories were collapsed to ease interpretation of data. To specify, for the purpose of this study, perpetrator was categorised as (1) stranger, (2) partner, friend, family, and (3) acquaintance, neighbour, another inpatient. Location was conceptualised as (1) home, or (2) street/elsewhere. Disclosure was conceptualised as (1) no one, (2) partner, family, friend, and (3) healthcare professional or police. The conceptualisations of these characteristics were based on our hypotheses.

### Data analysis

Analyses were performed in SPSS 22.0 [24]. Statistical significance was set at *p* < .05. To examine gender differences in demographic and clinical characteristics chi-squared test (in case of categorical variables) and independent t-tests (in case of continuous variables) were used. To examine gender differences in victimisation (threat, physical and sexual) in the past year or past 5 years, chi-squared tests were computed. Patients that had been a victim of threats, physical or sexual violence in the past 5 years were included in subsequent analyses. Three binary logistic regression models were run for threat, physical and sexual victimization incidents, with gender as the dependent variable and location, perpetrator and disclosure of the crime as independent variables. The models were run twice for each victimization type, to alternate the reference category for predictors with more than two categories (perpetrator and disclosure). Logistic regression models were corrected for significant confounders, which were identified in the descriptive analysis of demographic and clinical characteristics.

## Results

### Descriptive statistics

A total of 482 participants were included in this study, of which 105 (21.8%) were from the BEATVIC RCT study, 24 (5%) were part of the BEATVIC pilot study and 353 (73.2%) were part of the PHAMOUS-VICTROM study. Of the 482 participants, 170 (35.3%) were women. Demographic information per gender on the entire study sample can be found in Table 1. Women were significantly older than men. In comparison to women, a significantly greater proportion of men were diagnosed with schizophrenia as opposed to another psychotic disorder. There was no significant difference in gender for housing but compared to men, women were more likely to have no work. Further analyses were corrected for age, diagnoses and employment.

**Table 1:** Demographic information of study sample split by gender (*n* = 482)

	Men (*n* = 312)	Women (*n* = 170)	X^2^/t-value	*p*-value
Age (mean (SD))	44.28 (12.68)	49.13 (11.52)	−4.14	.001
Symptoms (PANSS, mean SD)				
Total	1.94 (.74)	1.84 (.71)	1.33	.19
Positive	1.93 (.92)	1.85 (.86)	0.93	.35
Negative	2.05 (.98)	1.96 (.96)	1.01	.32
General	1.72 (.77)	1.63 (.74)	1.13	.26
Diagnosis (n/%)				
Schizophrenia	198 (64.1%)	81(47.6%)		
Other psychotic disorder	111 (35.9%)	89 (52.4%)	12.17	<.001
Housing (n/%)				
Independent living	100 (40.3%)	61 (44.5%)	0.64	0.42
Supported housing	148 (59.7%)	76 (55.5%)		
Work (n/%)				
None	65 (31.3%)	60 (51.7%)	13.95	.001
< 12 h	66 (31.7%)	30 (25.9%)		
> 12 h	77 (37.0%)	26 (22.4%)		

### Prevalence victimization

In the past year, there were no differences between men and women in violent victimization (e.g., physical assault, threat with violence or with a weapon) and sexual victimization (e.g., forced sexual penetration, sexual touch without consent or sexual harassment) (see Table 2). However, over the past 5 years, men were more likely to have experienced threat with violence and were less likely to have experienced sexual victimization as opposed to women. For victimization of physical assault no significant differences between men and women in the past 5 years were found.

**Table 2:** Gender differences in threats, violent and sexual victimization

	Men (*n* = 111)	Women (*n* = 50)	X^2^	*p*-value
Victimization in the past year (n/%)				
Threat	22 (7.3%)	7 (4.3%)	1.62	.20
Physical	14 (4.7%)	2 (1.2%)	3.67	.06
Sexual	3 (1.0%)	5 (3.0%)	2.66	.10
Victimization in the past 5 years (n/%)				
Threat	62 (20.7%)	17 (10.5%)	7.68	.01
Physical	38 (12.7%)	11 (6.8%)	3.75	.05
Sexual	11 (3.6%)	22 (13.3%)	15.43	<.001

### Victimization characteristics

#### Incidents of physical threat

Although the counts and proportions imply that women were more likely to be threatened at home as opposed to in the street when compared to men (see Table 3), this risk was not significantly different from that of men after controlling for age, diagnoses and employment status. However, women were significantly more likely than men to be threatened by a partner/friend/family as opposed to strangers. For men the risk of being threatened by a stranger was higher than the threat coming from someone familiar. In comparison to men, women who experienced threats of physical violence were more likely to disclose to a professional/the police or to no one, instead of a partner/family/friend.

**Table 3:** Threats of physical violence: Counts, Proportions, and Logistic regression analyses with gender predicted by characteristics (location, perpetrator and disclosure) of the crime

	Men# (*n* = 62)	Women (*n =* 17)	OR (95% CI)^1^	OR (95% CI)^2^
Location	Count (%)	Count (%)		
Home_a_	23 (38.3%)	11 (64.7%)		
Street/Elsewhere	37 (61.7%)	6 (35.3%)	0.55 (0.08–3.93)	
Perpetrator				
Stranger_a_	25 (40.3%)	2 (11.8%)		0.02(.00–.63)*
Partner/ Friend/ Family_b_	19 (30.6%)	9 (52.9%)	52.49 (1.59–1737.72)*	
Acquaintance/ Neighbor/other inpatient	18 (29.0%)	6 (35.3%)	3.12 (0.28–35.08)	0.06 (.00–.11)
Disclosure				
No one_a_	10 (16.1%)	3 (17.6%)		30.38 (1.06–172.38)*
Partner/family/friend_b_	24 (38.7%)	5 (29.4%)	0.03 (0.01–0.95)*	
Professional/police	28 (45.2%)	9 (52.9%)	0.99 (0.09–11.20)	30.33 (1.09–840.91)*

**p < 0.01, *p < 0.05, # gender reference category (men), ^a^ reference category for first logistic regression, ^b^ reference category for second logistic regression, ^1^ Odds ratio for first logistic regression, ^2^ Odds ratio for second logistic regression (only for variables with 3 levels)

Analyses are corrected for age, diagnoses, and employment

#### Incidents of physical violence

The findings demonstrate that compared to men, women were significantly more likely to be victims of physical violence at home as opposed to on the street or elsewhere (Table 4). There were no significant differences between men and women where relationship with perpetrator or the choice to disclose was concerned. For the perpetrator category, the acquaintance/neighbor/patient option was compared to partner/friend/family, but not to stranger (due to small counts in cells). However, post-hoc chi-square analyses confirmed no significant differences between men and women where the type of perpetrator of the physical assault was concerned.

**Table 4:** Physical Violence: Counts and Proportions, and Logistic regression analyses, with gender predicted by characteristics (location, perpetrator and disclosure) of the crime

	Men# (*n* = 38)	Women (*n* = 11)	OR (95% CI)^1^	OR (95% CI)^2^
Location	Count (%)	Count (%)		
Home^a^	11 (29.7%)	6 (60.0%)		
Street/Elsewhere	26 (70.3%)	4 (40.0%)	0.06 (0.00–1.04)*	
Perpetrator				
Stranger^a^	13 (36.1%)	1 (9.1%)		n.a.
Partner/ Friend/ Family^b^	8 (22.2%)	4 (36.4%)	n.a.	
Acquaintance/ Neighbor/other inpatient	15 (41.7%)	6 (54.5%)	n.a.	0.75 (0.07–8.32)
Disclosure				
No one^a^	4 (10.8%)	2 (18.2%)		3.34 (0.06–198.11)
Partner/family/friend^b^	12 (32.4%)	1 (9.1%)	0.30 (0.01–17.78)	
Professional/police	21 (56.8%)	8 (72.7%)	3.55 (0.09–145.31)	11.85 (0.43–330.46)

**p < 0.01, *p < 0.05, # gender reference category (men), ^a^ reference category for first logistic regression, ^b^ reference category for second logistic regression, ^1^ Odds ratio for first logistic regression, ^2^ Odds ratio for second logistic regression (only for categories with more than 2 levels), n.a. not applicable due to small cell counts

Analyses are corrected for age, diagnoses, and employment

#### Incidents of sexual harassment or assault

The findings demonstrate a higher proportion of women becoming victims of sexual harassment or assault on the street or elsewhere, as opposed to at home (see Table 5), yet this pattern was not significant. There were no significant differences between male and female victims of sexual violence with regards to their relationship with the perpetrator nor with regard to disclosure of these kinds of incidents.

**Table 5:** Sexual Harassment or Assault: Counts and Proportions, and chi-squared analyses, with gender predicted by characteristics (location, perpetrator and disclosure) of the crime

	Men#	Women	OR (95% CI)^1^	OR (95% CI)^2^
Location				
Home^a^	5 (50.0%)	7 (35.0%)		
Street/Elsewhere	5 (50.0%)	13 (65.0%)	7.95 (0.64–984)	
Perpetrator				
Stranger^a^	2 (18.2%)	5 (23.8%)		1.87 (0.00–2035)
Partner/ Friend/ Family^b^	4 (36.4%)	8 (38.1%)	0.54 (0.00–583)	
Acquaintance/ Neighbor/other inpatient	5 (45.5%)	8 (38.1%)	0.04 (0.00–16.62)	0.07 (0.00–5.34)
Disclosure				
No one^a^	1 (10.0%)	3 (14.3%)		0.20 (0.00–48.33)
Partner/family/friend^b^	5 (50.0%)	9 (42.9%)	4.96 (0.02–1186)	
Professional/police	4 (40.0%)	9 (42.9%)	110 (0.05–2604)	22.39 (0.24–2056)

**p < 0.01, *p < 0.05, # gender reference category (men), ^a^ reference category for first logistic regression, ^b^ reference category for second logistic regression, ^1^ Odds ratio for first logistic regression, ^2^ Odds ratio for second logistic regression (only for categories with more than 2 levels),

Analyses are corrected for age, diagnoses, and employment

## Discussion

### Prevalence rate

The current study demonstrated that men diagnosed with a psychotic disorder were more likely to have experienced threats of physical violence than women, whereas women with a psychotic disorder were more likely to have experienced sexual harassment and assault in the past 5 years. Assessment of victimization in the past year shows a similar, yet non-significant trend. Overall, these findings are in line with patterns identified in the general population [7, 9]. One could argue that whereas studies from the general population conclude that there is a difference in prevalence for violent and sexual victimization between men and women, this difference might be smaller in people with a psychiatric disorder. It is possible that as victimization rates increase in individuals with a psychiatric disorder [1], the difference in prevalence of victimization types between men and women also fades and becomes less prominent.

### Characteristics of victimization: location

With regard to physical violence, women more often reported being victimized at home while men more often reported being victimized in the streets or elsewhere. This finding is also in line with studies examining victimization in the general population [7, 9]. Previous research found that men with a mental disorder more often used street drugs or alcohol when using violence themselves and women are more often violent at home (Robbins et al., 2003). Similarly, the use of street drugs might play a role in victimization of men in the streets. For threats with violence, a similar yet non-significant pattern of findings was found. As we suspected these findings on location (higher risk at home) to be (partly) correlated with the findings on the characteristics of the perpetrator (higher risks of being assaulted by partner than by a stranger) we performed a post-hoc test. This extra analysis demonstrated that location holds significantly different risks for men and women when added as a single predictor, but that this effect disappears (and is thus explained by) when type of perpetrator is added to the model. Other studies that did find a significant effect of location did not include multiple characteristics in the same model [7, 24] which is likely to explain the inconsistency between our study and previous research. Our findings indicate that location and perpetrator are closely linked violent victimization characteristics, and should be taken into account when tailoring personalized interventions.

For sexual assault and harassment, there were no significant differences in location between men in women. However, these results need to be interpreted with caution as only 3.6% of the men reported sexual victimization. Although it was hypothesized that women would be more likely victimized at home, there was a higher proportion of women (though not significant) who reported being sexually assaulted and/or harassed outside of their home. However, this pattern is in fact similar to the findings of a recent Dutch population survey, reporting that most victims (regardless of gender) were sexually assaulted or harassed outside the house, for example, in the streets or in a pub by someone from outside the domestic sphere [16]. It appears that on the basis of previous studies, that did not differentiate between victimization types; one would conclude that women are more likely to be victimized at home, where in fact this conclusion does not hold up for all types of victimization (i.e. violent victimization vs sexual victimization). More in depth research is needed to learn more about how characteristics of sexual victimization differ from characteristics of violent victimization or overall victimization.

### Characteristics of victimization: perpetrator

Regarding the type of perpetrator, men are more prone to be threatened by strangers whereas for women the risk is higher to be threatened by a person more familiar; these findings in our sample of people with a psychotic disorder are similar to findings in the general population [4, 5] and in patients with dual diagnosis [6]. Descriptive statistics showed similar results for actual physical violence although in formal testing this did not reach significance.

For sexual harassment and assault, no significant gender differences were found in type of perpetrator. Again, this seems to imply that characteristics of sexual victimization differ from violent victimization.

### Characteristics of victimization: disclosure

As expected there were no gender differences in disclosing the crime for actual violence and sexual assault or harassment. For threats with victimization, compared to men, women were less likely to disclose threat of physical violence to their partner, a friend or family than to disclose to a professional or the police, or to not disclose at all. Although this is not in line with our hypothesis that men and women do not differ in disclosure, our findings are likely explained by the fact that the partner, a friend or a family member in many cases was the perpetrator. This is also consistent with a previous study (CBS, 2015 [15]; that found no gender differences in reporting to the police in the general population, as in our sample men and women were just as likely to report to the police or a professional (45.2% vs 52.5%).

The current study has some limitations. First, victimization was examined with a self-report questionnaire, which is subject to memory bias. This could lead to an underreporting or overreporting of past incidences of victimization. However, a study on self-reports of violent victimisation in severe mental illness, showed that trauma history can be reliably assessed for research purposes [25]. Second, few victimization incidents were reported for the past year, and thus it was only possible to conduct formal analyses on victimization incidents of the past 5 years. Additionally, due to the low number of incidents some analyses may have been underpowered, yielding potentially biased estimates. Third, participants in this study were receiving treatment and were relatively stable in terms of symptoms at the time of assessment. It is likely that incidence rates of victimisation are higher in individuals with a psychotic disorder who are currently not in treatment. An important strength of the current study is the amount of detailed information, which enabled us to investigate gender differences in characteristics of different types of victimization in patients with a psychotic disorder for the first time.

## Conclusion and implications

In psychosis, gender patterns of victimization types and characteristics are similar to the general population. In men, violent victimization was more prevalent whereas women were more often sexually victimized. For violent victimization, men were more likely to become victimized in the streets or elsewhere by a stranger, whereas women were more often victimized at home by a partner, friend or a family member. There were no gender differences in characteristics of sexual victimization.

Although no gender differences in disclosure were found, the findings demonstrate that roughly half of victimization incidents are disclosed to health care professionals or police. Taking a closer look at our descriptive data, for all types of victimization 27 men disclosed to a professional only (50.9%), 20 men disclosed to the police only (37.7%) and six men disclosed to both (11.4%). For women, 14 disclosed to a professional only (54%), three disclosed to the police only (11.5%), and nine to both (34.5%). It is important for clinicians to be aware of signs of victimization, given that a) patients are more likely to become victimized than individuals in the general population, b) they may not disclose their victimization, and c) victimization is associated with severe symptomatology, increased use of psychiatric services, and poorer illness outcome [5].

For women with a psychotic disorder, clinicians may be more alert for signs of domestic and/or sexual abuse. Given that for violent victimization in women the perpetrator is often a family member, partner or friend, the clinician could make an additional effort to invite or involve the social network in treatment. Moreover, special attention may be paid to increasing assertiveness skills and conflict resolution skills for women specifically.

For men with a psychotic disorder, the risk of victimization is more present outside the home setting. Therefore, addressing this additional risk for men may involve a community approach, making use of the neighbourhood police and possibly launching anti-stigma campaigns. In treatment, specific attention may be paid to increasing ‘street skills’ (as described by an existing intervention for psychiatric patients targeting victimization [7];). Clinicians should also be aware of the fact that when men disclose their victimization, one third is disclosed to the police only, indicating for some men there might be a barrier to disclose to their clinician as well.

Future studies should investigate the possibility of tailoring interventions to patients with a psychotic disorder that are prone to different types of victimization in different locations and by different offenders. In addition, other aspects associated with different types of victimisation should be addressed in future studies, such as sexual orientation. Future studies may also investigate different locations (e.g. inpatient facility vs. outpatient housing) or differentiate further on specific perpetrators types (e.g. partner vs family member vs neighbour). Besides development of therapies directed at patients themselves, future interventions could put more emphasis on also including the community and the social network of the patient. Given that digital living is becoming the norm rather than the exception, future research into victimization of patients with a psychotic disorder should also include cybercrimes, such as sextortion.

## Data Availability

The datasets used and/or analysed during the current study are available from the corresponding author on reasonable request.
